# (Mis)Information, Fears and Preventative Health Behaviours Related to COVID-19

**DOI:** 10.3390/ijerph19084539

**Published:** 2022-04-09

**Authors:** Carmina Castellano-Tejedor, María Torres-Serrano, Andrés Cencerrado

**Affiliations:** 1 Psynaptic, Psicología y Servicios Científicos y Tecnológicos S.L.P., 08192 Barcelona, Spain; acence@gmail.com; 2 GIES Research Group, Basic Psychology Department, Autonomous University of Barcelona, 08192 Barcelona, Spain; 3 Research Group on Aging, Frailty and Care Transitions in Barcelona, Parc Sanitari Pere Virgili & Vall d’Hebron Research Institute (VHIR), 08023 Barcelona, Spain; 4 Faculty of Psychology, Autonomous University of Barcelona, 08192 Barcelona, Spain; torresserranomaria23@gmail.com

**Keywords:** COVID-19, pandemic, information needs, misinformation, preventative behaviours, online survey, cross-sectional survey, population mental health, anxiety, fear

## Abstract

Social and mass media platforms (SMM) are essential tools for keeping people informed about health-promoting practices. However, the potential to spread misinformation or false rumors exists. These might influence preventive health behaviours and incite anxiety and/or fear among the population. A sample of 300 adults participated in a survey to understand information needs, fears and preventive health behaviours related to COVID-19 while analyzing differences in COVID-19 acceptance rates. Descriptive-correlational, between-group comparisons and regression analyses were applied. Most of the sample revealed a willingness to accept COVID-19 vaccines (65.4% vs. 34.5%) and was prone to use and trust different SMM without experiencing significant obstacles in managing COVID-19-related information except for the need to ration it from time to time (χ^2^(2, *N* = 298) = 6.654, *p* = 0.036). Preventive behaviours/measures carried out were similar among the people resistant, hesitant or willing to get vaccinated for COVID-19. However, higher self-efficacy was observed in resistant vaccine individuals (F_(2)_ = 3.163, *p* = 0.044). Psychological impact (need for psychological support due to COVID-19 situation) in accepting (F_(5,_ _189)_ = 17.539, *p* < 0.001, R^2^ = 0.317) and hesitant individuals (F_(5,_ _77)_ = 17.080, *p* < 0.001, R^2^ = 0.526) was explained by female gender, younger age, threat susceptibility and differential characteristics in terms of psychological symptoms experienced and SMM trust. No explanatory model was obtained for the resistant individuals. SMM could be effective tools to promote COVID-19 health preventive behaviours. However, psychographic characteristics might modulate information-seeking and management as well as self-perceived threat susceptibility and severity. All these factors must be accurately considered when designing different health preventive campaigns for the general public.

## 1. Introduction

So far, millions of lives have been impacted by the COVID-19 pandemic [1]. However, scientific knowledge on how to effectively respond to COVID-19 outbreaks has also increased considerably in a very short time, and several research teams have worked intensively to develop COVID-19 vaccines. As a result, as of 20 July 2021, a total of 3,568,861,733 vaccine doses have been administered worldwide (WHO, 2021). Considering that the world population is approximately 7.7 million people [2], further reducing the COVID-19 burden relies on non-pharmacological interventions (NPIs). These COVID-19-related NPIs include lockdown policies, such as social restrictions and distancing, bans on nonessential travel, and the temporary closure of almost all businesses, facilities, and places of religious and other public gatherings, including funerals. According to epidemiological data in each country and region, some of these measures are still ongoing or are activated and deactivated to varying degrees. Other health regulations include quarantining symptomatic persons, tracking and following exposed individuals and health communication campaigns to increase the population’s awareness and acceptance of scientific knowledge related to COVID-19 spread and control.

In the current context of the COVID-19 pandemic, social and mass media (onwards SMM) have played a major role in acting as a very accessible information and social support source during the crisis [3]. In fact, a lot of professional and personal modern-day interactions are already conducted via phones and computers [4,5]. Thus, it is not a surprise that amid an unprecedented crisis for most of the population, the use of SMM might also rise, and people may consider it critically important for staying connected and updated. Also, government agencies and health institutions managing the crisis have used SMM to provide and dispense information, guidelines and regulations to the population [6]. Despite the many benefits of SMM, achieving greater reach and dispelling population inequalities regarding access to information, several downfalls must be considered.

First of all, there is a potential risk of using SMM to spread misinformation and false rumors [3,7] that might incite fear, as it did with Ebola [8] and to spur erroneous behavioral action [9]. In this sense, the World Health Organization (WHO) has opened a specific website to combat these COVID-related myths [10] and this overabundance of information (also known as an infodemic, as the WHO has precisely coined [11,12,13]). Also, many people might overly rely upon SMM in an attempt to regain self-perceived control and try to manage their anxiety [14]. Health information-seeking behaviour is most commonly described in the literature as an active or purposeful behaviour undertaken by an individual to find information about health [15,16]. The concept has been frequently studied in the context of chronic diseases. However, limited evidence exists in the context of a pandemic [17,18], and this behaviour may influence the self-protection measures that individuals take, and that, in turn, might modulate the anxious or fearful responses that they present to the stressor that motivates the search for information and support. In light of the potential for SMM to be used to self-regulate, express, and quell anxiety and fear (mainly through social support and information seeking as a coping mechanism), and even incite them (especially related to false information), it is important to examine the interaction between SMM use and psychological impact during times of crisis, as an effect that might influence vaccine acceptance among other health protective/preventive measures.

In this study, we applied a combination of the Health Belief Model (HBM) [19] and Protection Motivation Theory (PMT) [19,20] to examine potential factors for explaining COVID-19-related psychological impact among respondents. The HBM posits that perceived susceptibility, severity, and benefits are major contributors enabling individuals to take specific health behavioral actions. Moreover, the PMT posits that individuals’ health-related behaviours are triggered by their psychological distress (i.e., anxiety and fear). Therefore, the HBM and PMT theoretical frameworks might help understand what cues to action significantly influence protection/motivation behaviours (specifically, vaccination determination) and perceived self-efficacy concerning COVID contagion prevention. Apart from the HBM and PMT, the literature suggests the importance of obtaining accurate COVID-19 information from trusted sources. Indeed, misinformation concerning COVID-19 is associated with a greater fear of the disease [21,22] and fewer or less efficient protective behaviours [23].

**Figure 1:**
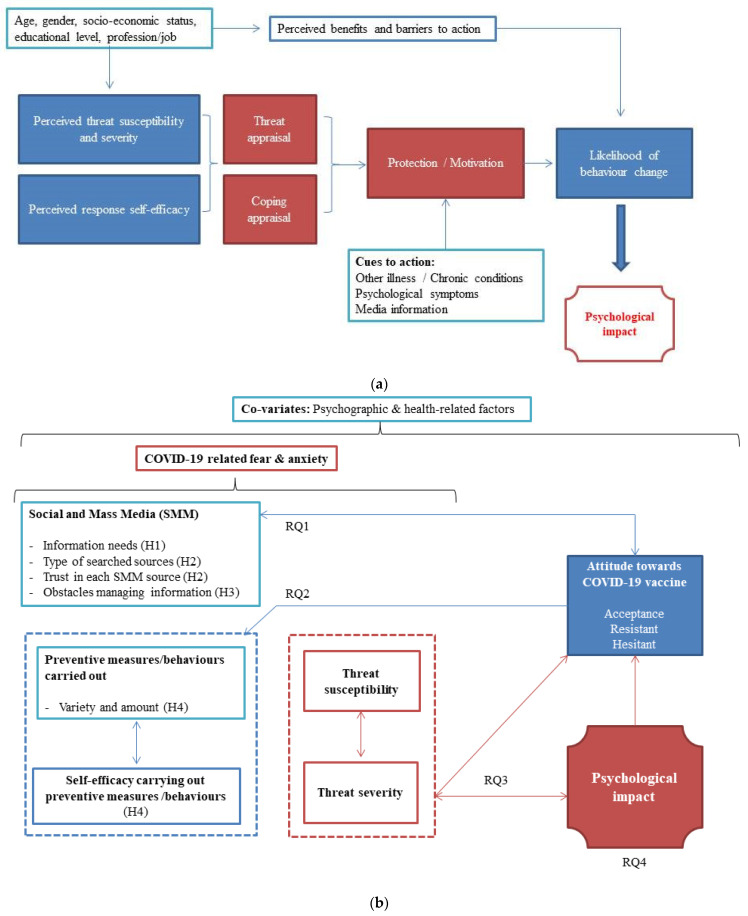
(**a**) Theoretical framework of the study combining HBM and PMT fundamentals [19,20] adapted for this research. (**b**) Framework map of the research questions (RQ) and hypothesis (H) of the study.

Figure 1a displays the theoretical framework of the study, and Figure 1b shows the relationship between the different factors explored in this study and the specific research questions (RQ) and hypothesis (H) analyzed in this correlational research:

RQ1: How do different profiles of individuals concerning attitudes towards COVID-19 vaccination (accepting, resistant, hesitant) behave in terms of SMM use to seek trustworthy information? And what type of obstacles related to COVID-19 information/management experience each of these profiles?

RQ2: How do different profiles of individuals concerning attitudes towards COVID-19 vaccination (accepting, resistant, hesitant) behave in terms of self-efficacy and preventive behaviours carried out?

RQ3: How perceived threat susceptibility and severity relate to psychological impact among different profiles of individuals concerning attitudes towards COVID-19 vaccination (accepting, resistant, hesitant)?

RQ4: How does psychological impact relate to SMM use/experience, self-efficacy and preventive behaviours carried out and for the different demographics and psychographic characteristics of the sample for each profile of individuals (accepting, resistant, hesitant)?

It is hypothesized that (H1) individuals accepting a COVID-19 vaccine will be those searching for greater amounts of information or having a greater need for information, (H2) those showing higher levels of trust in most of consulted SMM sources, (H3) those expressing fewer obstacles accessing and managing information, and also (H4) those carrying out a higher amount of preventive behaviours with medium-to-high self-efficacy. Similarly, and independently of the profile, (H5) higher threat susceptibility and severity will be related to the higher psychological impact.

## 2. Materials and Methods

### 2.1. Design and Recruitment

A web-based cross-sectional survey was executed recruiting a non-probabilistic sampling (convenience sampling) of community-based adults that utilize Spanish National Health Insurance. Inclusion criteria required being ≥18 years old and the only exclusion criteria was not understanding Spanish well enough to complete the survey. These criteria were stated in the informed consent presented before the survey. Additionally, questionnaires submitted below the cut-off of 4 min were excluded from the survey to ensure the reliability of the answers and to eliminate possible randomly completed surveys without reading and/or understanding the items.

### 2.2. Data Collection and Procedures

A 48-item multifactorial survey entitled “Personal Attitudes Towards the COVID-19 Pandemic” was conducted from 21 January to 21 March 2021 in the context of strict COVID-related gathering and mobility restrictions (via Kwik surveys platform, https://kwiksurveys.com), with three COVID-19 vaccines available on a voluntarily but highly recommended basis to all population in the country. The length of the survey was short enough not to take more than 10 min to ensure a high completion rate. This survey was distributed via mailing lists and social media (WhatsApp, Facebook and Twitter) from the authors’ accounts, and participants were encouraged to re-share it with their contacts and relatives. Potential participants, pre-accessing the survey, were informed about the research objectives and usefulness of results. The main ethical and privacy details were outlined as well, and permission to use the data for scientific purposes was requested before entering the survey. Participants could withdraw at any time before submitting their final responses. The Checklist for Reporting Results of Internet E-Surveys (CHERRIES) was employed (see Supplementary Material).

### 2.3. Outcomes and Covariates

#### 2.3.1. Primary Outcome Variable

The main outcome variable for this study was psychological impact. This variable was computed by analyzing participants’ responses to items 35 and 36 of the survey. Item 35 explored reasons for receiving psychological support among those respondents revealing that they were currently in treatment. Response options included: *previously existing problems, the COVID-19 situation, both options, other reasons*, and *prefer not to disclose*. Item 36 of the survey aimed to specifically evaluate the need for psychological support shortly due to COVID-19 health crises by answering if they will seek specific support for this matter. Scores ranged from 0 (*not likely at all*) to 10 (*extremely likely*). Two profiles (high psychological impact vs. medium-to-low psychological impact) were set up combining results from these two variables with those scoring ≥7 on item 36, and also those already receiving psychological support for COVID or both, due to pre-existing problems and the COVID-19 situation.

#### 2.3.2. Other Variables

Socio-demographic, medical and psychographic characteristics: participants’ socio-demographic data includes gender, age, residence area, nationality, cohabitation, employment status and educational level. Medical and psychographic characteristics were collected, including: suffering from a chronic disease, psychological symptoms, and if they have experienced losing a loved one due to COVID-19.

COVID-19-related data: participants indicated whether they have been diagnosed with COVID-19, the type of diagnostic test, the need for quarantine and/or hospital admission (including ICU). Participants were also asked about the degree to which they considered vaccines safe and effective (numeric scale ranging from 0–10) and the perception as the possible solution to end the pandemic caused by the COVID-19 (*yes* vs. *no* item).

COVID-19 preventive measures/behaviours and protection/motivation: Individual and voluntary preventative measures/behaviours were screened collecting the use of FPP2 masks, other masks, use of anti-COVID plastic face shields, transparent shields or other physical barriers on the desk/counter, hand hygiene with hydro-alcoholic gel, surface hygiene with hydro-alcoholic gel, wearing gloves, social distancing (1.5–2 m), restriction of departures at certain hours (curfew), limiting distance from home, limiting social gatherings to small groups, decreasing the frequency of attending to closed places, decreasing the frequency of attending crowded places for leisure and other actions. In addition, difficulties carrying out the different preventive measures/behaviours were also surveyed.

Compliance with the preventive measures (perceived response self-efficacy) was assessed through a self-reported 5-point numeric scale where 1 meant *lousy* and 5 *excellent* (item 5: *If you had to qualify your performance about the implementation of the different contagion prevention measures (own, recommended and/or imposed), what would you score?*).

Item 15 assessed personal predisposition and readiness of being vaccinated (protection/motivation) for COVID. This item read as follows: *Are you willing to be vaccinated for COVID-19?* For the purposes of this research, the sample was classified according to the following response options: (1) Accepting (*Yes*), (2) Hesitant (*Probably yes; I haven’t decided yet; Probably not; Depending on the vaccine*), (3) Resistant (*No, Just if I’m obliged*); Respondents stating *Do not know/Do not answer* (1%, *n* = 3), and *I have been already vaccinated* (16%, *n* = 48) were not included in these comparisons.

Perceived threat susceptibility and severity: The perceived threat was assessed through a 10-point Likert-scale with higher scores indicating greater threat susceptibility (item 2: *Currently, what is the degree of interference of the situation created by COVID-19 in your day-to-day life?*).

The perceived severity was assessed by means of a 10-point Likert-scale with a higher score indicating greater threat severity (item 3: *Comparatively with the situations you have experienced throughout your life, what personal severity does this COVID-19 pandemic situation have for you?*).

Fear of COVID–19 Scale (FCV-19S): The FCV–19S is a seven-item scale assessing the fear of COVID–19 using seven items rated on a 5-point Likert scale ranging from 1 (*strongly disagree)* to 5 (*strongly agree)*, with total scores ranging from 7 (minimum fear) to 35 (maximum fear) [24]. The present study demonstrated a reliability of α = 0.85.

Generalized Anxiety Disorder–7 (GAD-7): The GAD-7 is a seven-item self-report scale used in the medical and community settings to screen the severity of generalized anxiety [25]. Respondents are asked to answer how often they have been bothered by various anxiety symptoms over the last two weeks on a 4-point Likert scale ranging from 0 (*not at all)* to 3 (*nearly every day)*, with a total score ranging from 0 (*minimum anxiety*) to 21 (*maximum anxiety*). The present study showed the reliability of α = 0.90.

Social and Mass Media (SMM) Use: SMM use was measured exploring (1) the need for COVID-19 information (item 12: From the beginning of the pandemic to the present, what kind of information about COVID-19 have you actively sought via SMM? Point out everything that applies), (2) Sources of information and reliability (item 11: *What credibility do the different sources of SMM have for you when looking for COVID-19 related information (i.e., epidemiological data of infected, deceased, admissions, preventive measures, vaccine, etc.)? If you don’t use them regularly, please indicate the self-perceived credibility you judge they have*), and (3) Obstacles to COVID-19 information seeking (item 14: *What have been the main obstacles you have encountered in searching for or accessing information related to COVID-19? Point out everything that applies*).

### 2.4. Ethics

Research ethics procedures of this study complied with European and national legislation (e.g., the Charter of Fundamental Rights of the EU, Directive 95/46/EC of the European Parliament and of the Council of 24 October 1995 on the protection of individuals concerning the processing of personal data and on the free movement of such data) and data were treated with confidentiality, equality, and justice, respecting the Helsinki and the American Psychological Association (APA) ethical principles of research conduct. Codification procedures were employed to ensure the privacy and confidentiality of information. All participants were informed about study purposes, and direct informed consent was requested from all respondents before starting the survey and sending their responses. No economic incentives were offered for taking part in the survey. Participants who required feedback from the survey were invited to write down their email addresses and received information or specific helpful suggestions.

### 2.5. Statistical Analysis

Descriptive statistics were calculated for all primary and secondary outcome variables using measures of central tendency (mean, standard deviation, maximum/minimum range for continuous variables; frequencies and total percentages for categorical variables). For bivariate analysis, the Kolmogorov-Smirnov test was used to determine whether parametric or non-parametric tests were indicated. Bivariate comparisons were performed through either Student’s *t*-test and one-way ANOVA for variables with more than two categories or levels, the Mann-Whitney U test for continuous variables or the Chi-square test (and Fisher’s exact test when *n* < 5) for dichotomous variables. The correlation of two variables was compared using Pearson correlation two-sided, indicating significant correlations ≥0.3. The primary outcome variable describing the psychological impact of the COVID-19 pandemic was computed using scores on items 35 and 36 of the survey to assess if participants were currently in psychological treatment due to COVID-19 or if they believe they will need psychological treatment support in the near future due to the pandemic situation. Multiple regression analyses (stepwise) were employed to analyze the strength of the relationship between the primary outcome (as a continuous variable, item 36) and several predictor variables, as well as the importance of each of the predictors to the relationship (RQ5). The level of statistical significance was 5% (*p* ≤ 0.05). Bonferroni post hoc analyses were performed in all cases, and 95% confidence intervals were reported. All statistical analyses were performed using the SPSS_v25_ (IBM Corp., Armonk, NY, USA).

## 3. Results

### 3.1. Demographic Information and Response Rate

After excluding ineligible respondents (i.e., age below <18 years old or completing the survey in a very short time < 4 min), we finally collected 300 valid questionnaires (valid response rate was 93.45%) from adult respondents from different Spanish provinces (Barcelona 65.33%, *n* = 196). The majority of the sample lived in the region of Catalonia, with just a few cases being abroad (3%, *n* = 9) at the moment of the study. Sociodemographic characteristics are comprehensively presented in Table 1. Most respondents were women (75.7%, *n* = 227) with a mean age of 40.11 ± 12.34 years old (range 18–74), married or cohabiting with a partner (64%, *n* = 192) and in charge of vulnerable individuals (21.7%, *n* = 65) such as children, elderly and people with some physical and/or cognitive functional limitations.

The survey was closed after two months and a total of 300 completed responses had been attained. This was accepted by the researchers to run the statistical tests confidently during this challenging and sensitive time of the COVID-19 outbreak. An online survey was selected as the lockdown and enrollment of participants precluded in-person surveys and random selection.

### 3.2. Results Concerning RQ1: How Do Different Profiles of Individuals Concerning Attitudes towards COVID-19 Vaccination (Accepting, Resistant, Hesitant) Behave in Terms of SMM Use to Seek Trustworthy Information? And What Type of Obstacles Related to COVID-19 Information/Management Experience Each of These Profiles?

Table 2 displays different information needs referred by each profile of individuals regarding their willingness to accept a COVID-19 vaccine.

People willing to accept COVID-19 vaccines search for more information (F_(2)_ = 3.691, *p* = 0.026) concerning COVID-19 (*M* ± *SD* = 5.20 ± 2.32, 0–10, 95%CI [4.87, 5.53]) compared to the rest of the studied sample; both resistant (*M* ± *SD* = 4 ± 2.13, 1–10, 95%CI [3, 4.99]) and hesitant individuals (*M* ± *SD* = 4.66 ± 2.05, 1–9, 95%CI [4.21, 5.11]). However, these differences do not reach statistical significance in post hoc tests (H1).

As it can be observed, individuals willing to accept a COVID-19 vaccine were more prone to search for statistics related to COVID-19 spread, contagion index and mortality or other COVID-related epidemiological data compared to those hesitant or resistant to getting vaccinated (χ^2^ (2, *N* = 298) = 5.905, *p* = 0.005). Similarly, a higher tendency to search for information related to quarantine/confinement physical/psychosocial recommendations was observed among accepting individuals than the other subsamples (χ^2^ (2, *N* = 298) = 5.766, *p* = 0.056). Table 3 shows SMM use and experience for each subsample of studied individuals.

People willing to accept COVID-19 vaccines are more prone to use and trust information obtained via TV (F_(2)_ = 12.932, *p* < 0.001), the written press (F_(2)_ = 10.442, *p* < 0.001), WHO official channels (F_(2)_ = 8.858, *p* < 0.001) and National Health Department official channels (F_(2)_ = 11.347, *p* < 0.001) compared to those individuals hesitant or resistant to COVID-19 vaccination. Furthermore, they search for and trust more specialized COVID-19 related pages (F_(2)_ = 5.469, *p* = 0.005) (H2).

Similarly, accepting individuals tend to consult and trust to a higher degree information obtained from the radio (F_(2)_ = 9.086, *p* < 0.001), web-based media (F_(2)_ = 8.796, *p* < 0.001), healthcare professionals (F_(2)_ = 6.214, *p* = 0.002), scientific papers and publications (F_(2)_ = 6.018, *p* = 0.003), and regional health department official channels (F_(2)_ = 6.263, *p* = 0.002), compared to COVID-19 vaccine resistant individuals. Use and trust in these SMM were very similar between hesitant and resistant individuals (H2).

**Figure 2:**
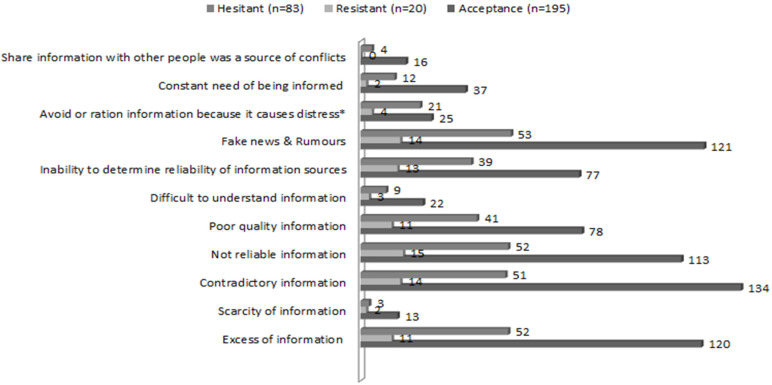
Obstacles to COVID-19 information-seeking (N = 300) ^a^. ^a^ Two missing values; * *p* < 0.05 according to the Chi-Square test.

Figure 2 displays obstacles to COVID-19 information seeking. Considering the whole sample, the most referred obstacles were contradictory information, fake news, rumours, excess and unreliable information.

There were no statistically significant differences in terms of obstacles related to COVID-19 information seeking between groups. In this sense, accepting individuals encountered an average of 3.87 ± 1.85 obstacles (0–9, 95%CI [3.61, 4.14]), resistant individuals encountered an average of 4.45 ± 1.99 obstacles (1–9, 95%CI [3.52, 5.38]), and hesitant individuals encountered an average of 4.06 ± 2.15 obstacles (0–9, 95%CI [3.59, 9.53]). However, individuals willing to accept a COVID-19 vaccine were more prone to avoid or ration information because they stated it might cause them distress compared to those individuals hesitant or resistant to get vaccinated (χ^2^(2, *N* = 298) = 6.654, *p* = 0.036) (H3).

For the whole sample, higher information-seeking behaviour was correlated with higher obstacles found when managing/searching for COVID-19-related information (*r* = 0.316, *p* < 0.001). Similarly, information-seeking behaviour was positively correlated with anxiety (*r* = 0.309, *p* < 0.001), as happened between anxiety and fear of COVID (*r* = 0.470, *p* < 0.001).

### 3.3. Results Concerning RQ2: How Do Different Profiles of Individuals Concerning Attitudes towards COVID-19 Vaccination (Accepting, Resistant, Hesitant) Behave in Terms of Self-Efficacy and Preventive Behaviours Carried Out?

**Figure 3:**
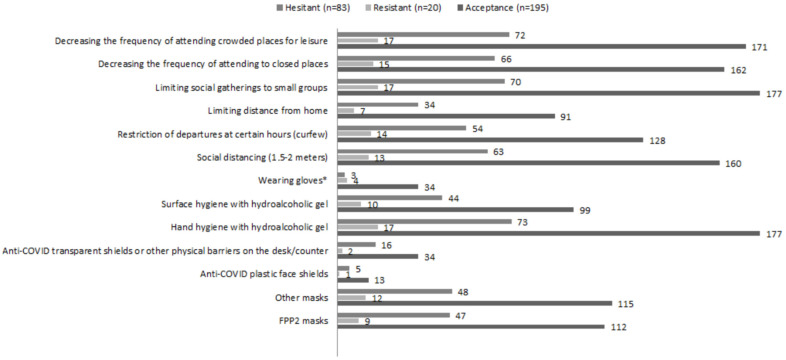
COVID-19 preventive measures/behaviors (N = 300) ^a^. ^a^ Two missing values; * *p* < 0.05 according to the Chi-Square test.

Self-efficacy complying and performing preventative measures (numeric scale 0–5) was significantly higher (F(2) = 3.163, *p* = 0.044) among resistant individuals (M ± SD = 4.20 ± 0.52; 95%CI [3.96–4.44]) compared to those accepting (*M* ± *SD* = 4.16 ± 0.62; 95%CI [4.08, 4.25]) or hesitant about (*M* ± *SD* = 3.96 ± 0.67; 95%CI [3.82, 4.11]) being vaccinated (H4). Figure 3 displays results concerning preventive measures/behaviours carried out for the sample.

There were no statistically significant differences among the three profiles in terms of attitudes/behaviours to prevent COVID except for wearing gloves (χ^2^ (2, *N* = 300) = 10.077, *p* = 0.006), with a higher percentage of people accepting being vaccinated (82,9%) carrying out this behaviour compared to 9.8% of resistant and 7.3% of hesitant individuals). However, this difference was because 26 out of 41 individuals wearing gloves worked as healthcare professionals and were within the subsample of people willing to get vaccinated. Overall, the average of behaviors/actions carried out to prevent contagion were similar between groups, despite accepting individuals showing a higher tendency to perform more actions (*M* ± *SD* = 7.55 ± 2.21, 0–13, 95%CI [7.24, 7.87]) compared to hesitant (*M* ± *SD* = 7.17 ± 2, 2–11, 95%CI [6.73, 7.60]) or resistant individuals, who tended to perform less actions (*M* ± *SD* = 6.90 ± 2.29, 1–10, 95%CI [5.83, 7.97]) (H4).

In this study sample, fear and anxiety did not significantly correlate with self-efficacy or preventive measures/behaviours carried out.

### 3.4. Results Concerning RQ3: How Perceived Threat Susceptibility and Severity Relates to Psychological Impact among Different Profiles of Individuals Concerning Attitudes towards COVID-19 Vaccination (Accepting, Resistant, Hesitant)?

The main outcome variable for this study was psychological impact (see Section 2.3.1), which was computed by analyzing participants’ responses to items 35 (reasons for receiving psychological support among respondents currently on treatment) and 36 (seek for psychological support due to COVID-19 health crises). Two profiles (high psychological impact vs. medium-to-low psychological impact) were set up combining results from these two variables with those scoring ≥7 on item 36 (33% highly distressed individuals, *n* = 99) and also those already receiving psychological support for COVID (4.3%, *n* = 13) or both, due to pre-existing problems and the COVID-19 situation (5%, *n* = 15). Thus, a final subgroup of 102 individuals (34%) experiencing high psychological impact due to COVID-19 was identified.

Table 4 displays results concerning threat susceptibility and severity for each profile of individuals depending on their degree of psychological impact due to COVID-19.

Overall, higher threat susceptibility was related to higher psychological impact (F_(1)_ = 11.756, *p* = 0.001). Also, threat severity was significantly associated with fear of COVID-19 (*r* = 0.414, *p* < 0.001).

Within groups, threat severity was higher among accepting individuals independently of psychological impact experienced (F_(2)_ = 3.397, *p* = 0.035), and statistically significant differences concerning threat susceptibility were observed between medium-to-low and high psychological distressed individuals willing to accept a COVID-19 vaccine (F_(1)_ = 9.519, *p* = 0.002) with the latest referring to higher threat susceptibility and, therefore, day-to-day interference (H5).

### 3.5. Results Concerning RQ4: How Psychological Impact Relates to SMM Use/Experience, Self-Efficacy and Preventive Behaviours Carried out and Different Demographics and Psychographic Characteristics of the Sample for Each Profile of Individuals (Accepting, Resistant, Hesitant)?

A regression model for accepting individuals was obtained (F_(5, 189)_ = 17.539, *p* < 0.001, R^2^ = 0.317), revealing that younger age, having a chronic condition, referring to higher threat susceptibility, anxiety and female gender were risk factors for experiencing higher psychological impact due to the COVID-19 situation in this subsample.

Also, a regression model for hesitant individuals was obtained (F_(5, 77)_ = 17.080, *p* < 0.001, R^2^ = 0.526), revealing that female gender, higher threat susceptibility and psychological symptoms experienced, lower trust in the Internet to obtained COVID-19-related information, but higher use (seeking, reading) of scientific papers in order to be informed, were revealed as significant factors contributing to a higher risk of suffering from psychological effects related to COVID-19 (Table 5).

## 4. Discussion

In this study, we addressed four research questions related to different profiles of individuals concerning attitudes toward vaccination (acceptance, resistance and hesitation). These were: (1) what information needs and level of trust in different social and mass media (SMM) sources present each profile of individuals, (2) how these different profiles behave in terms of preventive behaviours, (3) if so, how threat susceptibility and severity about the COVID-19 pandemic influenced psychological impacts and, finally, (4) what are (if any), the different relevant risk factors which might help to identify at risk individuals who suffer severe psychological effects as a result of the COVID-19 pandemic (Figure 1a,b)?

Overall, most respondents to this survey have accepted COVID-19 vaccines (65.4% of the studied sample). However, a non-negligible percentage of individuals revealed doubts about and resistance to vaccines. Despite advances in medicine and infectious disease treatments, it is noteworthy that, as the WHO has already stated in 2019, among the ten major threats in terms of public health, vaccine hesitancy is one of them. In this sense, a strengthening of anti-vaccination movements in recent decades has emerged, coinciding with unprecedented increases in the incidence of some communicable diseases (MERS, Influenza, etc.). Many intervention programs work from a deficit model of science communication, presuming that vaccination sceptics lack the ability to access or understand the evidence. Our study revealed no significant obstacles to understanding and accessing COVID-19 information (RQ1, H3), despite most referred obstacles being contradictory information, fake news, rumors, and an excess amount of non-reliable information regarding COVID. Also, individuals accepting a COVID-19 vaccine seemed to be those with more information needs (RQ, H1), and identifying and using higher amounts of trustworthy SMM (RQ1, H2), despite affirming that they also need to regulate and ration these information-seeking behaviours to avoid psychological distress (H3).

In the beginning and in the midst of the pandemic, some people might have perceived a low supply of evidence-based COVID-19 vaccine information, in paradoxical contrast with the vast amount of information circulating around the globe concerning COVID-19 [12,13]. This situation might have allowed the opportunity to generate and spread misinformation [26,27], which could have influenced the psychological impact on the general public. Therefore, interventions that target individual, community, cultural and societal-level factors are needed to protect people against rumors and conspiracy theories that flatten the misinformation curve and help health campaigns achieve their goals.

Concerning preventive behaviours, all profiles behave similarly, complying with most of the health preventive measures recommended by health institutions and authorities. Even if it is true that pro-vaccination individuals tend to undertake more (both self and hetero) protective measures and resistant individuals tend to less frequently undertake preventive behaviours, no significant differences were outlined (RQ2, H4). Interestingly, resistant individuals were those revealing higher self-efficacy committing with COVID-19 contagion prevention regulations, coinciding with less self-perceived severity and interference of COVID-19 in their day-to-day functioning (RQ2, H4). Most models of health-related behaviours are based on the assumption that people estimate the seriousness of a risk, that is to say, the possibility of loss based on the probability and severity of experiencing adverse outcomes, evaluating the costs and benefits of behaviour and then selecting a course of action that will maximize their expected outcome [28]. Despite not being able to confirm these hypotheses in our research due to the small sample size, it is possible that individuals resistant to COVID-19 vaccination were those who have been in less contact with the negative consequences of COVID (e.g., not experiencing losses, not caring for vulnerable people or suffering from chronic pathologies), and therefore, their perceived risk would be much lower compared to other individuals or experiencing higher optimistic risk perception [29,30]. In this line, our results found out that individuals perceiving COVID-19 as a higher threat were revealed as those more likely to search for psychological support, especially among those within the group of people that are pro-vaccination (RQ3, H5). Accordingly, and as expected, anxiety and fear of COVID strongly correlated between them and the latter with regard to threat susceptibility and severity [29].

Contrary to some previous research, the fear and anxiety induced by COVID-19-related threats do not lead our studied sample to carry out more health-related protective strategies [31]. However, information-seeking behaviours were positively associated with anxiety, indicating that independently of attitudes towards vaccination, those with higher arousal were also those more motivated to search for preventative behaviours/measures and information online [31].

One-third of the sample experienced psychological impact due to the COVID pandemic (34%). When trying to analyze significant predictors of psychological impact among the studied population, relevant risk factors identified were: female gender, younger age, having a chronic health condition, and already experiencing psychological symptoms altogether with threat susceptibility and low confidence in any SMM except for scientific papers and publications (RQ4). In addition, preliminary data have shown individuals at increased risk of clinically significant emotional distress, such as front-line COVID healthcare professionals, people with previous psychopathology, vulnerable populations or some specific profiles such as young women with someone vulnerable in charge [32,33,34,35]. In addition, at the beginning of the COVID-19 pandemic and in the absence of a vaccine or effective treatment, fear of COVID-19 and its consequences have left more people vulnerable to mental health problems, such as insomnia, anxiety, depression, posttraumatic stress, or even suicide in extreme cases [36,37]. Therefore, the importance of assessing psychological responses toward COVID-19 and its associated factors has been highlighted in the extant literature. More specifically, the adverse effects of elevated psychological distress may trigger information and reassurance-seeking behaviours, disorganized or inconsistent patterns regarding COVID-preventive measures, and compulsive-checking behaviours in response to potential threats of COVID-19 infection, in turn, may have an impact on the daily lives of individuals. However, the current scenario may be different in light of the impending vaccine. However, this fact can also cause distrust and fear in the population for the speed with which it has been developed.

Our findings revealed a clear profile of individuals with different self-protection motivations. Those willing to accept a COVID-19 vaccine presented higher information needs and use of SMM to seek a wide array of different COVID-19-related data. Most of them affirmed to search for statistics related to contagion rates, mortality and/or recommendations concerning quarantine, confinement and general self-care for physical and psychosocial realms during this health crisis. Furthermore, individuals willing to accept vaccination tend to consult and trust more SMM sources such as TV, the written press, the WHO, National Health Department web-based and official channels, and specialized COVID-related information sources compared to the rest of the population are hesitant or resistant to get vaccinated. However, they were also those experiencing higher threat susceptibility and severity concerning COVID-19. These might be related to a more straightforward coping mechanism to accept and keep contact with reality and act and decide on one’s health on an informed basis [15,16]. Our research has not found many individuals that are resistant to COVID-19 vaccination, and these were quite similar to the resistant ones. However, they could be precisely those with a higher risk of contagion or mid-long-term consequences due to COVID [38,39,40], and these profiles deserve further study.

### 4.1. Limitations

The results of our study should be considered in light of several limitations. Firstly, we selected the adult population (>18 years old) as the target age range of our non-probabilistic sample, and a high percentage of them reported middle-to-high socioeconomic status and held postgraduate or superior educational levels, 44% of them being healthcare professionals. Future studies are suggested to include larger representative samples from wide age ranges and lower-income or academic levels to facilitate the generalizability of findings. This is especially important in light of our findings and considering potential and relevant associations related to younger age [41] and female gender [42] steadily increasing. It is important to bear in mind that the response to this research was generated mostly from females (75.7%). This might be explained by the tendency of men to be more apathetic to a survey than women, which is consistent with previous research pointing out the same related to COVID-19 surveys [32] or other types of research [31,43]. Similarly, the very low proportion of resistant individuals (6.7%) undermines the ability to draw conclusions based on profiles. Also, this sample limitation could have been the cause of not finding any regression model to identify significant risk factors to experience psychological impact among individuals resistant to COVID-19 vaccines. In the same line, due to the nature of its design, this research does not allow us to infer cause-effect relationships. Finally, SMM use and experience is a vast study field. This research has only presented a preliminary and descriptive theoretical framework to start to depict potential relationships of its role in the context of severe health crises caused by COVID-19. The experience, needs and motivation of one’s SMM use require further investigation to better comprehend health behaviour studies in such contexts.

### 4.2. Implications

The COVID-19 pandemic led to unprecedented mitigation efforts that disrupted the daily lives of millions. Considering that traditional survey methods are time-consuming and expensive, we need timely and proactive data sources to respond to the rapidly evolving effects of health policy and the health crisis caused by COVID-19 on our population’s mental health. This research delves into describing and identifying the different profiles and attitudes of individuals related to COVID-19 vaccines. We have identified several main features of individuals willing to get vaccinated. However, those reluctant or not being sure of receiving a COVID-19 vaccine were more similar and revealed blurry profiles concerning the studied variables. Therefore, governments, public health officials, and advocacy groups must be prepared to address the issue of vaccine hesitancy and lower vaccine acceptance rates. An infodemic or, what could be worse, misinformation regarding the benefits, the medicinal composition, and the adverse effects of vaccination spread through multiple channels could have a considerable effect in terms of increased COVID-19 vaccine hesitancy [13]. Thus, we believe our results enrich the theoretical paradigm of health belief and protective models and the theoretical paradigm of public health management and health communication. Besides, different profiles of at-risk individuals who suffer from highly severe psychological effects due to COVID-19 have been identified, suggesting that more attention should be paid to younger female individuals with chronic conditions and those experiencing high threat susceptibility and, therefore, higher anxiety and psychological symptoms amid the pandemic. It would be interesting to consider using qualitative techniques for a more in-depth analysis involving this specific at-risk population to delve into this knowledge, which would probably help strengthen the dialogue with regard to the recent literature on this subject.

## 5. Conclusions

There is still a considerable size (34.5%) of the studied sample revealing doubts being reluctant to get vaccinated. In addition, this subsample of individuals displays a profile of low-information seeking behaviours, a tendency to find more obstacles when accessing and managing COVID-19 related information, and a certain distrust from official channels. In addition, despite carrying out similar preventive measures/behaviours to prevent COVID-19 contagion, vaccine hesitant individuals have higher self-efficacy in their performance compared to hesitant or COVID-19 vaccine accepting individuals. This emphasizes the need to carefully design and disseminate accurate and tailored health-prevention information campaigns when experiencing public health crises such as this one caused by COVID-19. Finally, the psychological impact is closely related to threat susceptibility and the latter, to threat severity and fear of COVID-19, as is significantly revealed in the subsample of people accepting COVID-19 vaccines. A profile of at-risk individuals who suffer psychological effects due to the COVID-19 situation was depicted, including females of younger age and those experiencing chronic conditions and a different range of psychological symptoms, including anxiety. This profile varies slightly depending on attitudes towards vaccination, and could serve to tackle and activate different mental health strategies to buffer the psychological impact this severe crisis could entail for the general population and especially for vulnerable individuals.

## Figures and Tables

**Table 1:** Participants’ demographics (*N* = 300).

Variables	*n* (%)
**Gender** (Female)	227 (75.7%)
**Country of origin**	
Spain	291 (97%)
Other ^a^	9 (3%)
**Cohabiting**	
Alone	32 (10.7%)
Couple/Partner	192 (64%)
Mother	57 (19%)
Father	40 (13.3%)
Children	118 (39.3%)
Siblings	26 (8.7%)
Grandmother	3 (1%)
Grandfather	1 (0.3%)
Mother-in-law	3 (1%)
Father-in-law	1 (0.3%)
Caregiver (formal/informal)	2 (0.7%)
Pet(s)	55 (18.3%)
Flat mates	16 (5.3%)
**Vulnerable individuals at charge** (Yes) ^b^	65 (21.8%)
**COVID-19 diagnosis** (Yes)	43 (14.3%)
**Chronic disease** (Yes)	55 (18.3%)
**Loss of a relative/family member due to COVID-19** (Yes)	51 (17%)
**Age**	
18–35	114 (38%)
36–59	160 (53.3%)
>60	26 (8.7%)
**Occupation** (Healthcare professionals vs. others)	131 (43.7%)
**Education**	
Primary school	9 (3%)
Secondary school	21 (7%)
Higher education	52 (17.3%)
University degree	178 (59.3%)
PhD	25 (8.3%)
Other degrees	15 (5%)
**Employment situation**	
Working	243 (81%)
Temporary Labor Force adjustment	5 (7.8%)
Dismissal	2 (3.1%)
Unemployed	9 (14.1%)
**Self-perceived socio-economic status**	
Low	52 (17.3%)
Medium	230 (76.7%)
High	18 (6%)
**Attitude towards COVID-19 vaccination**	
Accepting	195 (65.4%)
Resistant	20 (6.7%)
Hesitant	83 (27.8%)

^a^ This includes: UK (*n* = 2), Colombia (*n* = 2), Germany (*n* = 1), Norway (*n* = 1), Malaysia (*n* = 1), Indonesia (*n* = 1), Mexico (*n* = 1). ^b^ This includes elderly and young children (under 18 years old).

**Table 2:** Need for COVID information (*N* = 300) ^a,b^.

Variables (*yes* Option Displayed)	Accepting (*n* = 195) *n* (%)	Resistant (*n* = 20) *n* (%)	Hesitant (*n* = 83) *n* (%)
General information	167 (56%)	16 (5.4%)	70 (23.5%)
Statistical data *	126 (42.3%)	9 (3%)	43 (14.4%)
How to diagnose/identify positive cases	118 (39.6%)	11 (3.7%)	50 (16.8%)
Preventive/protective behaviors/measures	128 (43%)	9 (3%)	55 (18.5%)
COVID-19 treatment(s)	86 (28.9%)	8 (2.7%)	27 (9.1%)
Regulations/restrictions	165 (55.4%)	13 (4.4%)	68 (22.8%)
Quarantine/Confinement physical/psychosocial recommendations *	99 (33.2%)	5 (1.7%)	35 (11.7%)
Mental health recommendations	66 (22.1%)	3 (1%)	23 (7.7%)
Positive family functioning/environment recommendations	34 (11.4%)	3 (1%)	7 (2.3%)
Remote work and family life conciliation recommendations	25 (8.4%)	3 (1%)	9 (3%)
Other information searched:			
- Healthy lifestyles	1 (0.3%)	0	0
- COVID-19 courses/training	1 (0.3%)	0	0
- COVID-19 treatments for secondary effects	2 (0.6%)	0	0
- COVID-19 and pregnancy	1 (0.3%)	0	0
- COVID in the world	1 (0.3%)	0	1 (0.3%)
- COVID-19 vaccines	1 (0.3%)	0	0
- COVID-19 psychological effects	0	0	1 (0.3%)
- COVID-19 restrictions for funerals	1 (0.3%)	0	0
- COVID-19 data for educative system	1 (0.3%)	0	0

^a^ Two missing values; ^b^ Need for COVID-19 information was surveyed by means of item 12: From the beginning of the pandemic to the present, what kind of information about COVID-19 have you actively sought via SMM? Point out everything that applies); * *p* < 0.05 according to a chi-square test.

**Table 3:** Sources of Information and Reliability (*N* = 300) ^a^.

Variables	Accepting (*n* = 195) *M ± SD*, Range	Resistant (*n* = 20) *M ± SD*, Range	Hesitant (*n* = 83) *M ± SD*, Range
TV *	4.03 ± 1.29, 1–7	2.65 ± 1.35, 1–5	3.51 ± 1.30, 1–7
Radio *	3.90 ± 1.24, 1–7	2.75 ± 1.25, 1–5	3.54 ± 1.23, 1–6
Written press *	3.81 ± 1.23, 1–6	2.55 ± 1.23, 1–4	3.39 ± 1.39, 1–6
Web-based press *	3.79 ± 1.30, 1–7	2.55 ± 1.47, 1–5	3.43 ± 1.39, 1–6
Twitter	2.51 ± 1.37, 1–6	2.20 ± 1.44, 1–5	2.42 ± 1.05, 1–5
Facebook	1.93 ± 1.11, 1–6	2.10 ± 1.29, 1–5	2.18 ± 1.15, 1–6
Telegram	2.11 ± 1.17, 1–6	1.90 ± 1.55, 1–6	2.20 ± 1.08, 1–6
Whatsapp	1.92 ± 1.15, 1–7	2.05 ± 1.50, 1–7	2.14 ± 1.05, 1–5
Healthcare professionals *	5.69 ± 1.10, 3–7	4.85 ± 1.53, 2–7	5.35 ± 1.23, 1–7
Scientific papers & publications *	5.81 ± 1.18, 1–7	4.95 ± 1.57, 2–7	5.48 ± 1.06, 3–7
WHO official channels *	5.24 ± 1.33, 1–7	4.10 ± 1.74, 1–7	4.71 ± 1.44, 1–7
Health Department official channels (state) *	4.72 ± 1.34, 1–7	3.30 ± 1.78, 1–7	4.20 ± 1.47, 1–7
Health Department official channels (regional)*	4.67 ± 1.36, 1–7	3.60 ± 1.60, 1–7	4.34 ± 1.36, 2–7
Friends & acquaintances	3.04 ± 1.22, 1–7	2.50 ± 1.43, 1–5	3.01 ± 1.43, 1–7
The Internet (in general)	2.59 ± 1.09, 1–5	2.55 ± 1.60, 1–6	2.72 ± 1.13, 1–5
Specialized web pages *	4.41 ± 1.34, 1–7	3.65 ± 1.46, 1–6	3.95 ± 1.30, 1–7

^a^ Two missing values; * *p* < 0.05 according to one-way ANOVA tests with Bonferroni post-hoc comparisons.

**Table 4:** Threat susceptibility and severity in different profiles depending on their psychological impact (*N* = 300) ^a^.

	Accepting *(n* = 195) *		Resistant (*n* = 20)		Hesitant (*n* = 83)	
	Medium-to-Low Psychological Impact (*n* = 126)	High Psychological Impact (*n* = 69)	Medium-to-Low Psychological Impact (*n* = 14)	High Psychological Impact (*n* = 6)	Medium-to-Low Psychological Impact (*n* = 57)	High Psychological Impact (*n* = 26)
Threat susceptibility *M* ± *SD*, 95%CI, range	7.06 ± 2.20 [6.68–7.45] 0–10	7.99 ± 1.55 [7.61–8.36] 2–10	6.07 ± 2.40 [4.69–7.46] 2–9	7.50 ± 1.97 [5.43–9.57] 4–10	6.98 ± 1.67 [6.54–7.43] 2–10	7.31 ± 1.62 [6.65–7.96] 5–10
Threat severity, *M* ± *SD*, 95%CI, range	6.98 ± 2.12 [6.61–7.36] 0–10	7.41 ± 1.93 [6.99–7.87] 2–10	5.79 ± 2.81 [4.17–7.41] 0–10	6.83 ± 2.48 [4.23–9.44] 3–10	6.65 ± 2.17 [6.07–7.23] 1–10	6.42 ± 2.56 [5.39–7.46] 1–10

^a^ Two missing values; * *p* < 0,05 according to the One-Way ANOVA test.

**Table 5:** Multiple linear regression models obtained for psychological impact.

Accepting (*n* = 195)	B	*t*	*p*	95% CI
(Constant)		5.868	<0.001	4.125–8.302
GAD-7	0.286	4.583	<0.001	0.097–0.243
Age	−0.295	−4.810	<0.001	−0.100–−0.042
Threat susceptibility	0.171	2.796	0.006	0.074–0.430
Chronic disease	0.198	3.225	0.001	0.490–2.032
Gender	−0.181	−2.953	0.004	−2.043–−0.407
**Hesitant (*n* = 83)**	**B**	***t***	***p***	**95% CI**
(Constant)		0.663	0.509	−2.233–4.462
Total of psychological symptoms reported	0.451	5.359	<0.001	0.266–0.582
Trust in scientific papers & publication	0.243	2.970	0.004	0.206–1.043
Trust in Internet in general	−0.195	−2.462	0.016	−0.852–−0.90
Threat susceptibility	0.206	2.589	0.012	0.078–0.601
Gender	−0.265	−3.270	0.002	−2.869–−0.697

## Data Availability

The data that support the findings of this study are available from the corresponding author upon reasonable request.

## Supplementary Materials

The following supporting information can be downloaded at: https://www.mdpi.com/article/10.3390/ijerph19084539/s1, Table S1: The Checklist for Reporting Results of Internet E-Surveys (CHERRIES).
